# Isolation and Characterization of *ScGluD*2, a New Sugarcane beta-1,3-Glucanase D Family Gene Induced by *Sporisorium scitamineum*, ABA, H_2_O_2_, NaCl, and CdCl_2_ Stresses

**DOI:** 10.3389/fpls.2016.01348

**Published:** 2016-09-02

**Authors:** Yachun Su, Zhuqing Wang, Feng Liu, Zhu Li, Qiong Peng, Jinlong Guo, Liping Xu, Youxiong Que

**Affiliations:** Key Laboratory of Sugarcane Biology and Genetic Breeding, Ministry of Agriculture, Fujian Agriculture and Forestry UniversityFuzhou, China

**Keywords:** beta-1,3-glucanase, sugarcane-*Sporisorium scitamineum* interaction, defense response, adversity stimuli, expression profiles, agroinfiltration, antimicrobial action

## Abstract

Beta-1,3-glucanases (EC 3.2.1.39), commonly known as pathogenesis-related (PR) proteins, play an important role not only in plant defense against fungal pathogens but also in plant physiological and developmental processes. However, only a limited number of sugarcane beta-1,3-glucanase genes have been isolated. In the present study, we identified and characterized a new beta-1,3-glucanase gene *ScGluD*2 (GenBank Acc No. KF664181) from sugarcane. An X8 domain was present at the C terminal region of ScGluD2, suggesting beta-1,3-glucan-binding function. Phylogenetic analysis showed that the predicted ScGluD2 protein was classified into subfamily D beta-1,3-glucanase. Localization of the ScGluD2 protein in the plasma membrane was determined by tagging it with green fluorescent protein. The expression of *ScGluD*2 was more up-regulated in sugarcane smut-resistant cultivars in the early stage (1 or 3 days) than in the susceptible ones after being challenged by the smut pathogen, revealing that *ScGluD*2 may be involved in defense against the invasion of *Sporisorium scitamineum*. Transient overexpression of *ScGluD*2 in *Nicotiana benthamiana* leaves induced a defense response and exhibited antimicrobial action on the tobacco pathogens *Pseudomonas solanacearum* and *Botrytis cinerea*, further demonstrating that *ScGluD*2 was related to the resistance to plant pathogens. However, the transcripts of *ScGluD*2 partially increased (12 h) under NaCl stress, and were steadily up-regulated from 6 to 24 h upon ABA, H_2_O_2_, and CdCl_2_ treatments, suggesting that ABA may be a signal molecule regulating oxidative stress and play a role in the salt and heavy metal stress-induced stimulation of *ScGluD*2 transcripts. Taken together, *ScGluD*2, a novel member of subfamily D beta-1,3-glucanase, was a stress-related gene of sugarcane involved in plant defense against smut pathogen attack and salt and heavy metal stresses.

## Introduction

Plants generate pathogenesis-related (PR) proteins in response to pathogen infection. Beta-1,3-glucanase (EC 3.2.1.39), a well-known example of PR proteins, is widely distributed in higher plants (Leubner-Metzger, 2012). As reported, the disease-resistant effect of beta-1,3-glucanase can catalyze the hydrolytic cleavage of beta-1,3-glucans, a major structural component present in the fungal cell wall, and inhibit the growth of pathogens (Chen et al., 2006; Shi et al., 2006; Singh et al., 2014). Additionally, its hydrolysate oligosaccharide can be used as an elicitor to induce a chain of systemic resistance in plants, such as promoting the generation of many PR proteins and defense-related products (Chen et al., 2006; Shi et al., 2006; Singh et al., 2014). So far, various beta-1,3-glucanase genes from *Arabidopsis thaliana* (Escobar et al., 2003), *Oryza sativa* (Romero et al., 1998), *Triticum aestivum* (Kemp et al., 1999; Liu et al., 2010), and *Zea mays* (Jondle et al., 1989) have been induced by pathogen attack.

In addition to the proposed role in defense response, beta-1,3-glucanase may also be involved in diverse plant physiological and developmental processes, such as seed and pollen germination (Morohashi and Matsushima, 2000; Wan et al., 2011), bud dormancy (Rinne et al., 2001), flower growth and fruit ripening (Akiyama et al., 2004; Tao et al., 2013). The expression of beta-1,3-glucanase genes has been shown to be regulated by certain environmental stresses, such as pathogen infection (Gu et al., 2008; Liu et al., 2010), wounding (Wu and Bradford, 2003), salt (Su et al., 2013), and plant hormone stimuli (Akiyama and Pillai, 2001; Wu and Bradford, 2003; Liu et al., 2010). Liu et al. (2010) detected the beta-1,3-glucanase gene, *TaGlu*, in wheat that was induced by the stripe rust pathogen *Puccinia striiformis* f. sp. *tritici*, salicylic acid (SA), methyl jasmonate (MeJA), and ethylene (ET). Gu et al. (2008) demonstrated that the overexpression of tobacco chitinase I gene and the beta-1,3-glucanase gene in sugarcane (*Saccharum* spp.) had different inhibitory efficiencies on the growth of the sugarcane smut pathogen (*Sporisorium scitamineum*). Wu and Bradford (2003) found that the *GluB* gene showed a tissue-specific regulation in tomato seeds and leaves, and its gene expression level was slightly up-regulated by MeJA and wounding during tomato seed germination. Akiyama and Pillai (2001) have reported that the expression of an endo-1,3-beta-glucanase (*OsGLN1*) from rice was up-regulated by abscisic acid (ABA) and drought stress.

Beta-1,3-glucanases with multiple structural isoforms and are divided into four subfamilies (A, B, C, and D) according to molecular size, isoelectric point (pI), primary structure, cellular localization, and expression patterns (Romero et al., 1998). Thirteen beta-1,3-glucanase genes, among which nine were classified into subfamily A (*Gns2*, *Gns3*, *Gns4*, *Gns5*, and *Gns6*), subfamily B (*Gns1*), subfamily C (*Gns7* and *Gns8*) and subfamily D (*Gns9*) according to their structure and function, have been identified from the rice genome (Romero et al., 1998). Six highly similar endo-beta-1,3-glucanase genes (*TaGlb2a*, *TaGlb2b*, *TaGlb2c*, *TaGlb2d*, *TaGlb2e*, and *TaGlb2f*) from wheat clustered within subfamily A were cloned and characterized. These six *TaGlb2* genes were phylogenetically related to each other and were differentially regulated during development processes and in response to powdery mildew (*Erysiphe graminis*) and head blight (*Fusarium graminearum*) pathogens (Higa-Nishiyama et al., 2006). Shi et al. (2006) isolated two beta-1,3-glucanase genes (*FaBG2-2* and *FaBG2-3*) from strawberry and observed different expression levels of these two genes under *Colletotrichum fragariaeor* and *C. acutatum* infection.

In our previous study, two sugarcane beta-1,3-glucanase genes *ScGluA1* (GenBank Acc No. KC848050, subfamily A) and *ScGluD1* (GenBank Acc No. KC848051, subfamily D) were detected from sugarcane post-inoculation with *S. scitamineum* (Su et al., 2013). We have also validated that both genes were located in the apoplast. Analysis of prokaryotic expression and gene expression patterns analysis indicated that *ScGluA1* showed positive response to biotic and abiotic stimuli; however, *ScGluD1* did not (Su et al., 2013). In this report, a new sugarcane beta-1,3-glucanase subfamily D gene, *ScGluD*2 was cloned and identified from the smut-resistant cultivar Yacheng05-179 infected by *S. scitamineum* for 2 days. Its phylogenetic features, subcellular localization, and expression profiles in sugarcane against *S. scitamineum* and different chemicals stimuli were analyzed. In addition, the transient expression of *ScGluD*2 in *Nicotiana benthamiana* was further investigated by conductivity measurement, DAB (3,3′-diaminobenzidine) staining, the detection of immunity associated marker genes expression, and the pathogen infection test according to a previous study (Choi et al., 2012), which reflect the function of ScGluD2 protein. This study aims to obtain a better understanding of the role of *ScGluD*2 in response to various adversity stresses.

## Materials and Methods

### Plant Materials and Treatments

Nine sugarcane cultivars including eight newly released and one of the most popular cultivar ROC22 in mainland China, were selected for the investigation of *ScGluD*2 transcripts under *S. scitamineum* stress. The smut whips and sugarcane plants were obtained from the Key Laboratory of Sugarcane Biology and Genetic Breeding, Ministry of Agriculture (Fuzhou, China). Two-bud setts of nine sugarcane cultivars (Supplementary Table S1), including four smut-resistant (YZ03-258, YZ01-1413, YT96-86, and LC05-136), three medium susceptible (GT02-467, ROC22, and FN39) and two susceptible (YZ03-103 and FN40) cultivars, which were previously investigated in the field by scientists at the China Agricultural Research System (personal communication with Yingkun Huang), were inoculated with a smut spore suspension at a concentration of 5 × 10^6^ spores/mL (containing 0.01% Tween-20, v/v). Those inoculated with sterile distilled water served as the control (Que et al., 2014a). The punctured material was cultured at 28°C in a photoperiod of 16 h light/8 h dark. After inoculation, the sugarcane buds were excised at 0, 1, 3, and 7 days. Collected samples were frozen in liquid nitrogen and stored at -80°C.

The uniform 4-month-old sugarcane tissue culture plantlets of the smut-resistant cultivar Yacheng05-179 (private bulletin) were used for the following seven different stress treatments in conical tubes at 28°C in a photoperiod of 16 h light/8 h dark (Su et al., 2014a), and three biological replicates with three plantlets for each were carried out. The plantlets were treated with either 5 mM SA, 25 μM MeJA, 100 μM ABA, 10 mM hydrogen peroxide (H_2_O_2_), or 25% polyethylene glycol (PEG) 8000 following the procedures described by Su et al. (2014a), respectively. Then the entire sugarcane plantlets were sampled at 0, 6, 12 and 24 h, respectively. For salt or heavy metal treatment, plantlets were treated with 250 mM sodium chloride (NaCl) or 500 μM cadmium chloride (CdCl_2_), and sampled at 0, 12, 24, and 48 h (Su et al., 2014a). Collected samples were frozen in liquid nitrogen and stored at -80°C until further analysis.

### RNA Extraction and cDNA Synthesis

Total RNA was extracted using TRIZol Reagent (Invitrogen, Carlsbad, CA, USA) according to the manufacturer’s protocol. Isolated RNA was treated with DNase I (Promega, Madison, WI, USA) to eliminate the residual DNA. Then, 1 μg RNA in a final reaction volume of 20 μL was used for first-strand cDNA synthesis with the Prime-Script^TM^ RT Reagent Kit (TaKaRa, Dalian, China) using random hexamer primers following the manufacturer’s instructions.

### Cloning and Sequence Analysis of a *ScGluD*2 Gene from Sugarcane

Due to the lack of whole-genome sequencing of sugarcane, the cloning primers ScGluD2-cDNAF and ScGluD2-cDNAR (**Table 1**) for the putative sugarcane beta-1,3-glucanase-encoding gene *ScGluD*2 were designed according to the sequence information of *Sorghum bicolor* beta-1,3-glucanase gene (GenBank Acc No. Sb02g037380; Paterson et al., 2009). The cDNA sample of the Yacheng05-179 buds after *S. scitamineum* inoculation for 2 days as above was used for the amplification of the putative beta-1,3-glucanase gene. The amplified fragment was cut from the gel and ligated into the pMD18-T vector (TaKaRa, Dalian, China) and sequenced (Shenggong, Shanghai, China).

**Table 1:** Primers used in this study.

Primer	Sequence (5′–3′)	Strategy
ScGluD2-cDNAF	GCACAAGGATATGTCGTC	RT-PCR
ScGluD2-cDNAR	ATGCTTTACATCACTACAAATAGAA	RT-PCR
ScGluD2-QF	TATTGCTGTGGGTAATGAGGTCC	qRT-PCR
ScGluD2-QR	TTGAAAGTGGCAGCAGAGGGAG	qRT-PCR
GAPDH-QF	CACGGCCACTGGAAGCA	qRT-PCR
GAPDH-QR	TCCTCAGGGTTCCTGATGCC	qRT-PCR
ScGluD2-SublocF	TGCTCTAGAATGTCGTCCAAGAGACTACA	Subcellular localization vector construction
ScGluD2-SublocR	GGACTAGTCATCACTACAAATAGAATGGGC	Subcellular localization vector construction
ScGluD2-1301F	ATAAGAATGCGGCCGCATGTCGTCC	Overexpression vector construction
ScGluD2-1301R	CGGGATCCTTACATCACTACAAATAGAATG	Overexpression vector construction
NtHSR201F	CAGCAGTCCTTTGGCGTTGTC	qRT-PCR
NtHSR201R	GCTCAGTTTAGCCGCAGTTGTG	qRT-PCR
NtHSR203F	TGGCTCAACGATTACGCA	qRT-PCR
NtHSR203R	GCACGAAACCTGGATGG	qRT-PCR
NtHSR515F	TTGGGCAGAATAGATGGGTA	qRT-PCR
NtHSR515R	TTTGGTGAAAGTCTTGGCTC	qRT-PCR
NtNPR1F	GGCGAGGAGTCCGTTCTTTAA	qRT-PCR
NtNPR1R	TCAACCAGGAATGCCACAGC	qRT-PCR
NtPR-1a/cF	AACCTTTGACCTGGGACGAC	qRT-PCR
NtPR-1a/cR	GCACATCCAACACGAACCGA	qRT-PCR
NtPR2F	TGATGCCCTTTTGGATTCTATG	qRT-PCR
NtPR2R	AGTTCCTGCCCCGCTTT	qRT-PCR
NtPR3F	CAGGAGGGTATTGCTTTGTTAGG	qRT-PCR
NtPR3R	CGTGGGAAGATGGCTTGTTGTC	qRT-PCR
NtEFE26F	CGGACGCTGGTGGCATAAT	qRT-PCR
NtEFE26R	CAACAAGAGCTGGTGCTGGATA	qRT-PCR
NtAccdeaminaseF	TCTGAGGTTACTGATTTGGATTGG	qRT-PCR
NtAccdeaminaseR	TGGACATGGTGGATAGTTGCT	qRT-PCR
NtEF1αF	TGCTGCTGTAACAAGATGGATGC	qRT-PCR
NtEF1αR	GAGATGGGGACAAAGGGGATT	qRT-PCR

The deduced amino acid sequence and open reading frame (ORF) of the *ScGluD*2 gene were searched with the ORF Finder program^1^. ProtParam^2^, SignalP 4.1 Server^3^, TMHMM Server v. 2.0^4^, NetNGlyc 1.0 Server^5^, and SMART^6^ programs were used to predict the physical and chemical parameters, the presence and location of signal peptide cleavage sites, the transmembrane helices, the N-glycosylation sites and the conserved domains in amino acid sequences of ScGluD2, respectively. Multiple alignments of the amino acid sequences were done using the NTI software. A phylogenetic tree of ScGluD2 together with ScGluA1 and ScGluD1 and the beta-1,3-glucanases from three other plant species, was generated using the neighbor-joining method and a bootstrap of 1,000 replicates with MEGA 5.05 software application (Liu et al., 2010; Su et al., 2013).

### Gene Expression Patterns of *ScGluD*2 under Various Adversity Stresses

The expression of *ScGluD*2 under *S. scitamineum*, SA, MeJA, ABA, H_2_O_2_, PEG, NaCl, and CdCl_2_ stresses was evaluated with quantitative real-time PCR (qRT-PCR). The *ScGluD*2-specific PCR primers ScGluD2-QF and ScGluD2-QR (**Table 1**) were designed by Primer Premier 5.0 software. Primers GAPDH-QF and GAPDH-QR (**Table 1**) derived from the glyceraldehyde-3-phosphate dehydrogenase (*GAPDH*) gene were used as an internal control according to previous researches conducted by our group (Que et al., 2009; Ling et al., 2014). PCR efficiencies of *ScGluD*2 and *GAPDH* were determined by the standard curve method (Schmittgen and Livak, 2008; Supplementary Figure S1). The qRT-PCR was performed with FastStart Universal SYBR Green Master (ROX) Kit (Roche, China) on a 7500 real time PCR system (Applied Biosystems, Carlsbad, CA, USA). The reaction mixture contained 12.5 μL FastStart Universal SYBR Green PCR Master (ROX), 2.0 μL template (100 × diluted cDNA), and 0.4 μM of each primer in a 25 μL total volume. The conditions were as follows: 50°C for 2 min; 95°C for 10 min; 40 cycles at 95°C for 15 s, and 60°C for 1 min. The melting curve was then obtained at the end of each reaction (95°C for 15 s, 60°C for 1 min, and 95°C for 15 s) to verify the specificity of the PCR product. The expression level of the target gene was quantified from three replicates using the 2^-ΔΔCt^ method (Livak and Schmittgen, 2001). Statistical analysis was conducted using the Data Processing System (DPS) v7.05 software (China). Data were expressed as the mean ± standard error (SE). Significance (*p*-value < 0.05) was calculated using one-way analysis of variance (ANOVA) followed by multiple Duncan tests. For *S. scitamineum* stress, the relative expression of the target gene was calculated by the inoculation expression level minus the control level at each corresponding time point.

### Subcellular Localization Assay

The PSORT Prediction program^7^ was used for the prediction of ScGluD2 subcellular localization. The ORF fragment of *ScGluD*2 without stop codon was amplified by the primer pairs of ScGluD2-SublocF and ScGluD2-SublocR (**Table 1**) followed by insertion into the *Xba*I and *Spe*I sites of the pCAMBIA 2300-*GFP* expression vector. The positive recombinant pCAMBIA 2300-*ScGluD*2-*GFP* was confirmed by PCR and sequencing and then transformed into *Agrobacterium tumefaciens* strain EHA105. The assay for *Agrobacterium*-mediated transient expression in *N. benthamiana* cells was performed according to our previously published method (Su et al., 2014a). After cultivation at 24°C (16 h light/8 h darkness) for 2 days, the GFP fluorescence in *N. benthamiana* leaves was observed using fluorescence microscopy (Axio Scope A1, Zeiss, Oberkochen, Germany).

### *Agrobacterium*-Mediated Transient Overexpression of *ScGluD*2 in *N. benthamiana*

The ORF coding of *ScGluD*2 was amplified by the primer pairs of ScGluD2-1301F and ScGluD2-1301R (**Table 1**) and then ligated into the *Xba*I and *Bam*HI sites of the pCAMBIA 1301 overexpression vector. The positive recombinant pCAMBIA 1301-*ScGluD*2 was checked by PCR and sequencing, followed by transformation into *Agrobacterium* EHA105. The assay for *Agrobacterium*-mediated transient overexpression in *N. benthamiana* leaves was conducted as described previously (Su et al., 2014b, 2015). After injection and incubation at 24°C (16 h light/8 h darkness), the measurement of ion conductivity and DAB staining in the agroinfiltrated leaves were carried out at 1 and 2 days, respectively (Su et al., 2014b). Meanwhile, the expression of the *ScGluD*2 gene as well as nine tobacco immunity associated marker genes including the hypersensitive response (HR) marker genes *NtHSR201*, *NtHSR203*, and *NtHSR515*, the SA associated gene *NtNPR1*, the jasmonic acid (JA) associated genes *NtPR-1a/c*, *NtPR2*, and *NtPR3*, and the ET synthesis-dependent genes *NtEFE26* and *NtAccdeaminase* (primers listed in **Table 1**), were analyzed by qRT-PCR after 24 h of infiltration (Su et al., 2014b). *NtEF1α* gene (primers listed in **Table 1**) was used as an internal control (Schmidt and Delaney, 2010; Su et al., 2014b) and its PCR efficiency was calculated following the standard curve method (Schmittgen and Livak, 2008; Supplementary Figure S1).

### Expression of *ScGluD*2 in *N. benthamiana* Plants in Response to Pathogen Infection

The EHA105 strains carrying the pCAMBIA 1301-*ScGluD*2 or the empty vector pCAMBIA 1301 were diluted in MS liquid medium (containing 200 μM acetosyringone) to OD_600_ = 0.8 and then infiltrated into the eight-leaf stage-old *N. benthamiana* leaves. After incubation at 28°C (16 h light/8 h darkness) for 1 day, the cultured tobacco pathogen cells (OD_600_ = 0.5) of *Pseudomonas solanacearum* or *Botrytis cinerea*, which were diluted in 10 mM MgCl_2_, were infiltrated into the main vein of the infected leaves, respectively. All treatment materials were cultivated at 28°C (16 h light/8 h darkness) for 20 days and photographed (Que et al., 2014a).

## Results

### Cloning and Sequence Analysis of the Sugarcane Acidic Subfamily D Beta-1,3-glucanase (*ScGluD*2) Gene

Reverse transcription-polymerase chain reaction (RT-PCR) with primers designed based on the gene sequence of *S. bicolor* beta-1,3-glucanase (GenBank Acc No. Sb02g037380) resulted in a full-length putative sugarcane beta-1,3-glucanase cDNA which was named as *ScGluD*2 (GenBank Acc No. KF664181). The whole sequence of the *ScGluD*2 gene was 1,500 bp nucleotides with an intact ORF (from 11 to 1,495 bp) encoding 494 amino acid residues. Its calculated molecular mass and pI were 52.78 kDa and 5.84, respectively. SignalP 4.1 Server prediction results showed that the most likely cleavage site of ScGluD2 occurred between positions 22 and 23. One transmembrane helix domain was predicted between positions 7 and 29. Three potential N-glycosylation sites, NVSG, NITY, and NFTG, were observed at the positions 19, 109, and 255, respectively.

**FIGURE 1:**
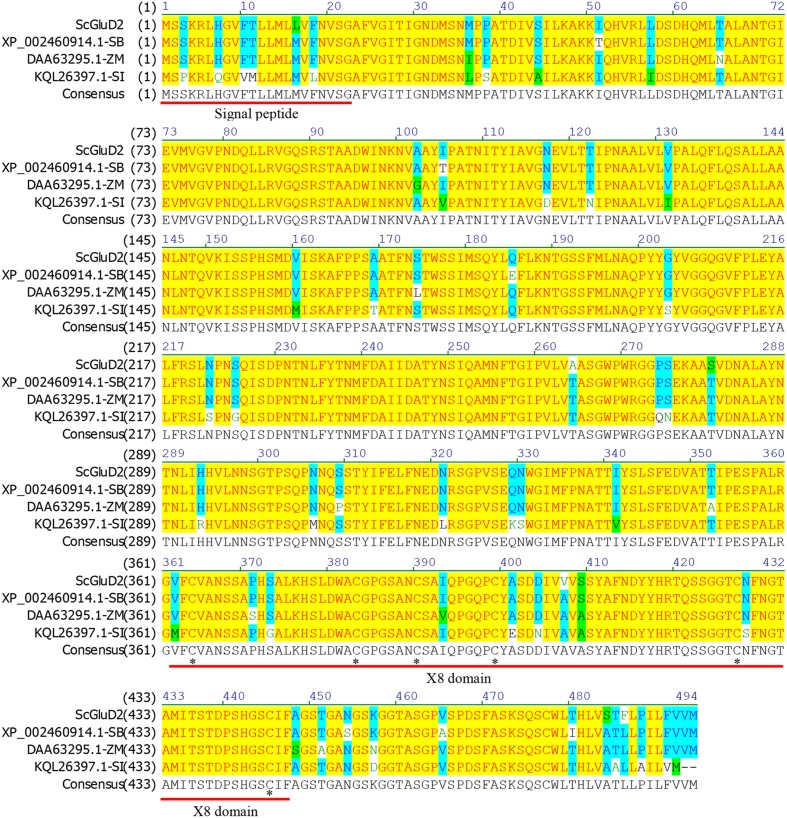
**Amino acids sequence alignment of ScGluD2 and other plant beta-1,3-glucanaes by the NTI program.** The predicted signal peptide and X8 domain are underlined. The asterisks indicate a conserved distribution of six cys residues in the X8 domain. SB, *Sorghum bicolor*; ZM, *Zea mays*; SI, *Setaria italica*.

**FIGURE 2:**
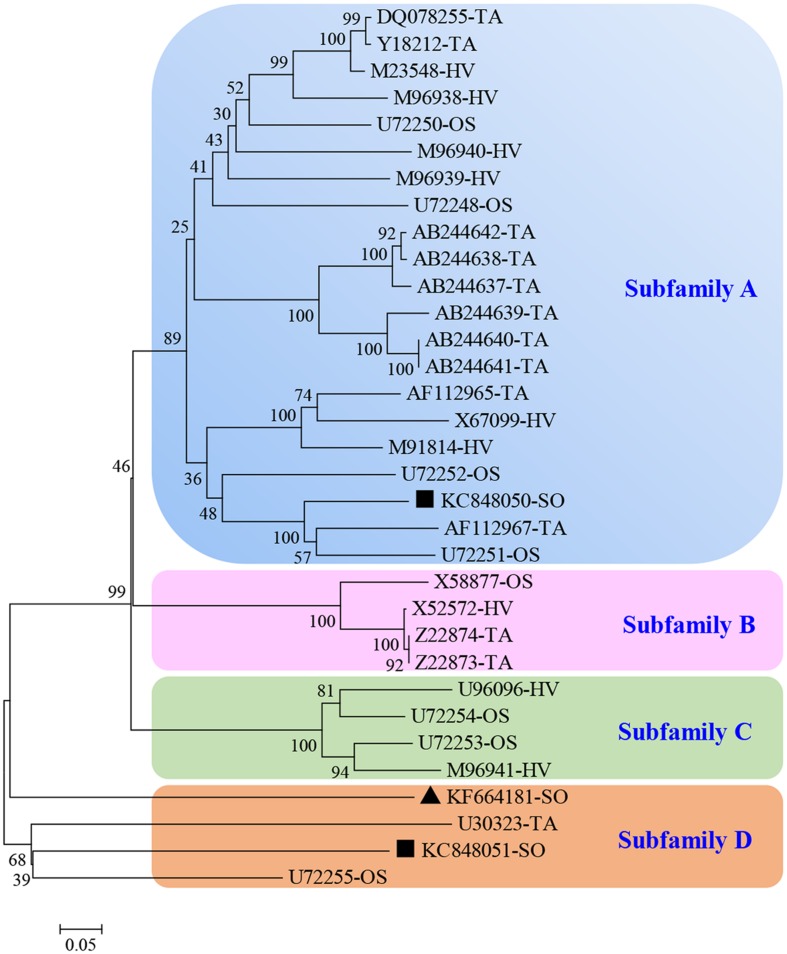
**Phylogenetic tree of ScGluD2 and other plant beta-1,3-glucanaes.** The un-rooted tree was constructed using the Neighbor–Joining method by the MEGA 5.05 program. Bootstrap values indicate the divergence of each branch and the scale indicates branch length. GenBank accession numbers (species from which the beta-1,3-glucanase originated follows it) are shown in the tree. TA, *Triticum aestivum*; HV, *Hordeum vulgare*; OS, *Oryza sativa*; SO, sugarcane. Scale bar, 0.05 substitutions per site. The bootstrap support values are shown on the branches. ▲KF664181-SO, ScGluD2; ■KC848050, ScGluA1; ■KC848051, ScGluD1.

After BLASTP analysis, the identity of the deduced amino acid sequence of *ScGluD*2 with known beta-1,3-glucanases from *S. bicolor* (GenBank Acc No. XP_002460914.1), *Z. mays* (GenBank Acc No. DAA63295.1), and *Setaria italica* (GenBank Acc No. KQL26397.1) was 98, 96, and 91%, respectively (**Figure 1**). Furthermore, a signal peptide between positions 1 and 22, a glycosyl hydrolase family 17 conserved domain between positions 25 and 346, and an X8 domain (containing six cys residues) between positions 362 and 447 were predicted in the amino acid sequences of ScGluD2 and the other three beta-1,3-glucanases (**Figure 1**). A phylogenetic tree was constructed with the putative amino acid sequences of *ScGluD*2 and beta-1,3-glucanases from other plant species. As shown in **Figure 2**, four major subfamilies were distinguished, which was in well-accordance with previous studies (Liu et al., 2010; Su et al., 2013). ScGluD2 was clustered within subfamily D and was closely related to the previously characterized beta-1,3-glucanases from *T. aestivum* (GenBank Acc No. U30323), sugarcane (GenBank Acc No. KC848051), and *O. sativa* (GenBank Acc No. U72255; **Figure 2**). However, the ScGluD2 protein only showed 18.67 and 24.30% identity with sugarcane *ScGluA1* and *ScGluD1* at the amino acid sequence level, respectively. All of these findings implied that *ScGluD*2 was a new subfamily D member of sugarcane beta-1,3-glucanase.

### Detection of *ScGluD*2 Transcripts in Nine Sugarcane Cultivars Post-inoculation with *S. scitamineum*

**FIGURE 3:**
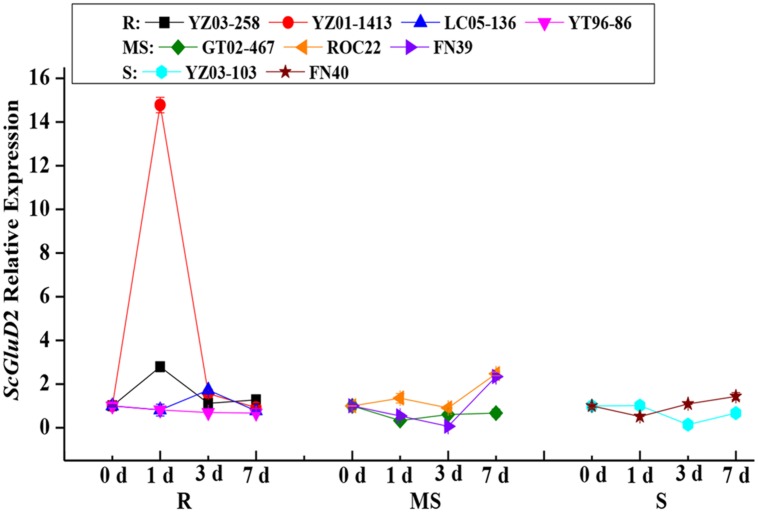
***ScGluD*2 transcripts in nine sugarcane cultivars post-inoculation with *Sporisorium scitamineum*.** The tested sugarcane samples including four smut-resistant (YZ03-258, YZ01-1413, YT96-86, and LC05-136), three medium susceptible (GT02-467, ROC22, and FN39) and two susceptible (YZ03-103 and FN40) cultivars. The *ScGluD*2 transcripts were calculated by the expression level of the inoculated sample minus the level of the mock at each corresponding time point, so as to eliminate any effect of wounding. R, resistance; MS, moderate susceptibility; S, susceptibility. qRT-PCR data were normalized to the *GAPDH* expression level. Error bars represent standard error.

To assess the role of the *ScGluD*2 gene in sugarcane defense against fungal infections, qRT-PCR analysis was performed to examine the *ScGluD*2 transcripts in the buds of nine different sugarcane cultivars after being challenged by *S. scitamineum* (**Figure 3**). Among the four smut-resistant cultivars, the *ScGluD*2 transcripts in YT96-86 nearly maintained at a stable level from 0 to 7 days, and it is noteworthy that the transcripts of *ScGluD*2 in YZ03-258, YZ01-1413, and LC05-136 were markedly increased by 2.79-, 14.77-, and 1.72-fold as early as 1 or 3 days and then returned to the control level at 7 days. However, in three medium susceptible (GT02-467, ROC22, and FN39) and two susceptible (YZ03-103 and FN40) cultivars, *ScGluD*2 was observed to be decreased or remained unchanged from 0 to 3 days on the whole, followed by being up-regulated and reaching a peak value at 7 days. These results revealed that *ScGluD*2 may be a positive responsive component of smut resistance in sugarcane.

### Gene Expression Patterns of *ScGluD*2 under SA, MeJA, ABA, H_2_O_2_, PEG, NaCl, and CdCl_2_ Stresses

**FIGURE 4:**
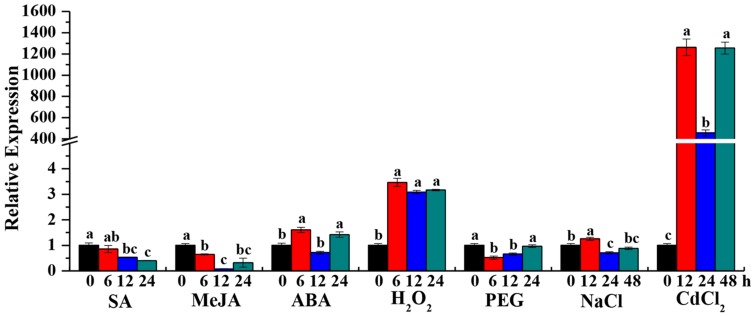
**Gene expression patterns of *ScGluD*2 under various abiotic stresses by qRT-PCR analysis.** The samples of 4-month-old Yacheng05-179 plantlets, which used for qRT-PCR analysis, are treated by 5 mM SA, 25 μM MeJA, 100 μM ABA, 10 mM H_2_O_2_, 25% PEG 8000, 250 mM NaCl, and 500 μM CdCl_2_, respectively. Expression level of *GAPDH* is used as internal control. All data are represented as means of three replicates (*n* = 3) ± SE. Different lowercase letters indicate a significant difference (*p*-value < 0.05) compared to the control as determined by the least-significant difference test. SA, salicylic acid; MeJA, methyl jasmonate; ABA, abscisic acid; H_2_O_2_, hydrogen peroxide; PEG, polyethylene glycol; NaCl, sodium chloride; CdCl_2_, cadmium chloride.

Beta-1,3-glucanase has been shown to be induced by external stimuli in several plant species (Wu and Bradford, 2003; Liu et al., 2010). To determine whether the *ScGluD*2 transcripts can be stimulated by adverse environment, the expression patterns of *ScGluD*2 in response to the plant hormone stresses of SA, MeJA, and ABA, oxidative stress of H_2_O_2_, hyper-osmotic stresses of PEG and NaCl, and heavy metal stress of CdCl_2_ were characterized by qRT-PCR (**Figure 4**). Compared to the control, *ScGluD*2 showed an inhibited expression pattern under SA and MeJA stresses. In the case of ABA treatment, the transcripts of *ScGluD*2 were significantly up-regulated and reached the peak value at 6 and 24 h at about 1.60- and 1.42-fold higher than the control, but remained unchanged at 12 h. When sugarcane plantlets were exposed to H_2_O_2_, *ScGluD*2 transcript increased significantly at 6 h (3.46-fold) and remained stable until 12 h (3.08-fold) and 24 h (3.16-fold). Under the PEG 8000-induced drought simulation condition, *ScGluD*2 mRNA level was significantly reduced to 1.93- and 1.51-fold at 6 and 12 h, and then increased to the expression level of the control at 24 h. For NaCl treatment, *ScGluD*2 transcript levels were significantly induced at 12 h (1.25-fold), while decreased at 24 and 48 h as compared with the control. Furthermore, the expression pattern of *ScGluD*2 under heavy metal stress (CdCl_2_) was detected. Interestingly, *ScGluD*2 transcript levels sharply increased from 12 to 48 h under CdCl_2_ treatment, increasing up to 1,260.22-, 455.18-, and 1,253.54-fold of that of the control. These results suggest that *ScGluD*2 is a stress-related gene, which positively responded to ABA, H_2_O_2_, NaCl, and CdCl_2_ stimuli in sugarcane but was suppressed during SA, MeJA, and PEG treatments.

### Subcellular Localization Assay

**FIGURE 5:**
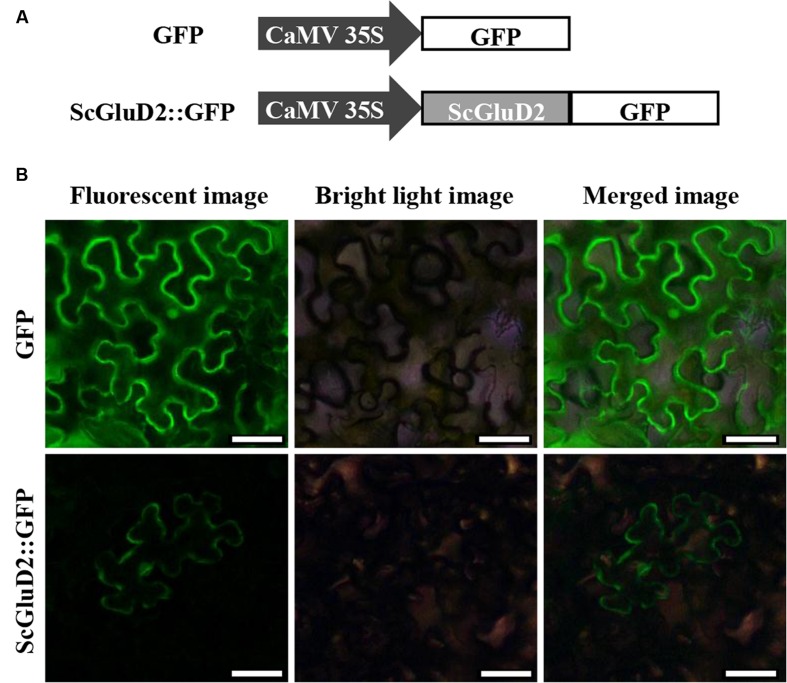
**Subcellular localization of ScGluD2 protein. (A)** The structures of ScGluD2::GFP and GFP control vector. **(B)** Subcellular localization of ScGluD2::GFP fusion protein in *Nicotiana benthamiana* leaves 48 h after infiltration. Bar = 50 μm.

To identify the subcellular localization of the ScGluD2 protein, the target gene fused with the *GFP* reporter gene in the pCAMBIA 2300 vector was constructed (**Figure 5A**). After being transiently expressed in *N. benthamiana* leaves for 48 h, ScGluD2::GFP fluorescence localized to the plasma membrane was visualized (**Figure 5B**). Conversely, the control GFP was distributed throughout the cell (**Figure 5B**). This result was consistent with the sequence analysis that ScGluD2 was predominantly localized to the plasma membrane with a probability of 64% using the Psort predicted program.

### Induction of Defense Response by Transient Overexpression of *ScGluD*2 in *N. benthamiana*

**FIGURE 6:**
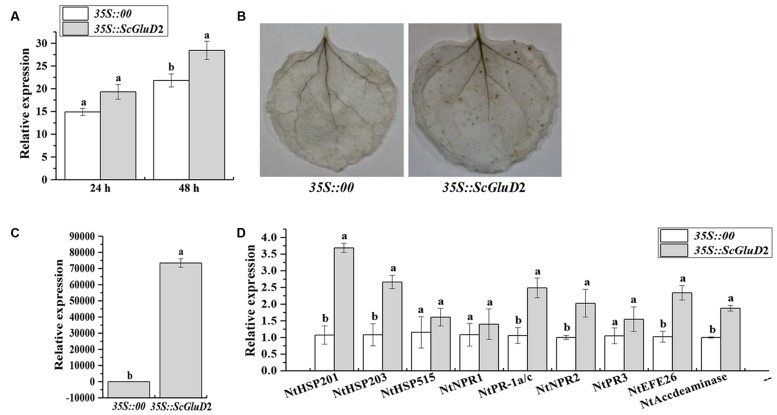
**Transient overexpression of *ScGluD*2 in *Nicotiana benthamiana* leaves infiltrated with *Agrobacterium* EHA105 carrying *35S::00*- (control) or *35S::ScGluD*2 construct. (A)** Conductivity measurement of *35S::ScGluD*2-transiently expressing leaves at 24 and 48 h after agroinfiltration. **(B)** DAB (3,3′-diaminobenzidinesolution) staining of leaf tissues 48 h after infiltration. **(C)** Relative expression level of the *ScGluD*2 gene in *35S::ScGluD*2-transiently expressing leaves at 24 h after agroinfiltration. **(D)** Relative expression level of the immunity associated marker genes in *N. benthamiana* after infiltration for 24 h, including the hypersensitive response (HR) marker genes *NtHSR201*, *NtHSR203* and *NtHSR515*, the salicylic acid (SA) associated gene *NtNPR1*, the jasmonic acid (JA) associated genes *NtPR-1a/c*, *NtPR2* and *NtPR3*, and the ethylene (ET) synthesis-dependent genes *NtEFE26* and *NtAccdeaminase.* Expression level of *NtEF1α* is used as the internal control. All data are represented as mean of three replicates (*n* = 3) ± SE. Different lowercase letters indicated a significant difference (*p*-value < 0.05) compared to the control, as determined by the least-significant difference test.

A BLASTP comparison of the ORF region of *ScGluD*2 showed no similarity with any *N. benthamiana* genes and only 39.41, 52.71, and 52.99% amino acid sequence identity with the glucan endo-1,3-beta-glucosidase from *N. tabacum* (GenBank Acc No. XP_016472663.1), *N. sylvestris* (GenBank Acc No. XP_009778460.1) and *N. tomentosiformis* (GenBank Acc No. XP_009631831.1) in GenBank. For the constraints faced by sugarcane transformation, to partly determine whether the protein function of *ScGluD*2 was involved in defense response, *Agrobacterium*-mediated transient overexpression in *N. benthamiana* was performed. Within 24–48 h after agroinfiltration, leaves transiently expressing *35S::ScGluD*2 exhibited enhanced (1.30-fold) ion conductivity compared with the control (**Figure 6A**). At 48 h, the DAB polymers were detected in the leaves that overexpressed *ScGluD*2; this H_2_O_2_ accumulation suggests that an HR occurred (**Figure 6B**). However, the control was free of DAB staining (**Figure 6B**). Additionally, the transcripts of *ScGluD*2 (**Figure 6C**) as well as several defense-related genes (**Figure 6D**), including the HR marker genes *NtHSR201* and *NtHSR203*, the JA associated genes *NtPR-1a/c* and *NtPR2*, and the ET synthesis-dependent genes *NtEFE26* and *NtAccdeaminase*, were increased 72,183.68-, 3.44-, 2.46-, 2.35-, 2.02-, 2.28-, and 1.88-fold in *ScGluD*2-expressing leaves over that of the control, respectively. These data indicated that the expression of the *ScGluD*2 gene was correlated with the plant defense response.

### Expression of *ScGluD*2 in *N. benthamiana* Plants in Response to Pathogen Infection

**FIGURE 7:**
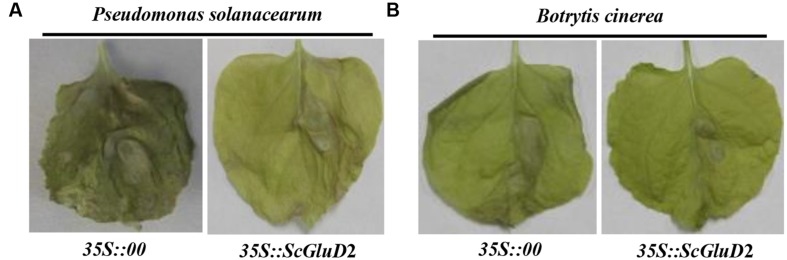
**Infection results of *Nicotiana benthamiana* leaves by *Pseudomonas solanacearum* (A) and *Botrytis cinerea* (B) after infiltration with *35S::00*- (control) or *35S::ScGluD*2-containing *Agrobacterium* strain.** Disease symptoms of infected leaves were observed at 20 days post-inoculation with *P. solanacearum* and *B. cinerea*, respectively.

To further test the inhibitory effect of the *ScGluD*2 gene, *Agrobacterium* EHA105 strains carrying the vector of pCAMBIA 1301 (control) or pCAMBIA 1301-*ScGluD*2 constructs were transiently expressed in the *N. benthamiana* leaves for 1 day, followed by infiltration with tobacco pathogens. As shown in **Figure 7**, a distinct disease symptom was observed in *N. benthamiana* leaves of the control (*35S::00*) 20 days after inoculation with *P. solanacearum* (**Figure 7A**) or *B. cinerea* (**Figure 7B**). In contrast, the leaves infiltration with *35S::ScGluD*2 did not show more severe disease symptoms than those of the control. Our data, therefore, suggest that *ScGluD*2 expression showed an antimicrobial action on *P. solanacearum* and *B. cinerea* in *N. benthamiana*.

## Discussion

Sugarcane accounts for 92% of the total sugar production in China. Sugarcane smut, which is caused by *S. scitamineum*, occurs widely in sugarcane planting areas and has become one of the most difficult fungal diseases to control (Sundar et al., 2012; Que et al., 2014b). To date, a limited number of disease resistant genes have been characterized in sugarcane. Beta-1,3-glucanase is known to be a typical PR2 protein which could be induced by pathogenic infection and plays a role in plant defense response (Cheong et al., 2000; Leubner-Metzger, 2012). In plant genomes, beta-1,3-glucanases are encoded as relatively large gene families which can be divided into four classes (Dobnik et al., 2013). The major achievement of this study was the isolation and identification of a novel beta-1,3-glucanase gene *ScGluD*2 from sugarcane. The cDNA of *ScGluD*2 has a complete ORF that encoded beta-1,3-glucanase protein of 494 amino acids. As reported, the X8 module is found at the C terminus of family 17 glycosyl hydrolases of beta-1,3-glucanase (Henrissat and Davies, 2000). This domain is characterized by a conserved distribution of six cys residues and a phe residue and is possibly involved in carbohydrate binding (Simpson et al., 2009). Similarly, the X8 domain with a 6Cys-box was present at the C terminal region of ScGluD2, suggesting beta-1,3-glucan-binding function.

Previous reports have noted an increase in the expression of various beta-1,3-glucanases in plants during pathogenic infection (Kemp et al., 1999; Liu et al., 2010; Su et al., 2013). Kemp et al. (1999) observed that the activity of beta-1,3-glucanase was induced in three near-isogenic wheat lines after being infected with *P. recondita* f. sp. *tritici*. A higher level of beta-1,3-glucanase activity was also detected in infected than in the non-infected wheat plant by Western blot analysis (Kemp et al., 1999). Sugarcane buds serve as the invasion route of smut pathogen. Spore germination occurs on the sugarcane internodal surface and then forms the appressoria on the inner scales of young buds (Sundar et al., 2012). After the teliospore deposition 6–36 h, *S. scitamineum* enters into the bud meristem (Alexander and Ramakrishnan, 1980; Sundar et al., 2012). It is worth mentioning in the present study that the elevated expression levels of *ScGluD*2 in sugarcane smut-resistant cultivars were presented in the early stage (1 or 3 days) more than in the susceptible ones (**Figure 3**), suggesting that *ScGluD*2 may participate in the resistance of sugarcane against *S. scitamineum* infection. This was similar to the findings reported by Liu et al. (2010), in which the transcripts of *TaGlu* gene were much higher in the incompatible interaction than in the compatible interaction between wheat and *P. striiformis* f. sp. *tritici*. Our previous study also demonstrated that beta-1,3-glucanase activity in the resistant sugarcane cultivar (Yacheng05-179) increased faster and lasted longer than that of the susceptible one (Liucheng03-182), and the transcripts of *ScGluA1* and *ScGluD1* were up-regulated and slightly down-regulated post-inoculation with *S. scitamineum*, respectively (Su et al., 2013). Although from the same subfamily D (**Figure 2**), the opposite responses to *S. scitamineum* stress between *ScGluD*2 and *ScGluD1* implied there might be a functional diversity in the sugarcane beta-1,3-glucanase multigene family. Similarly, two highly homologous beta-1,3-glucanase genes, *FaBG2-2* and *FaBG2-3*, were identified from strawberry, while the gene expression level of *FaBG2-3* was remarkably higher than that of *FaBG2-1* under *C. fragariaeor* and *C. acutatum* stresses (Shi et al., 2006).

In this study, *ScGluD*2 was induced by smut pathogen infection in sugarcane, which in turn warrants an investigation on the role of its encoding gene in further transient expression. The overexpression of *ScGluD*2 in *N. benthamiana* (**Figures 6** and **7**) indicated that it may be involved in plant defense. Levine et al. (1994) reported a close relationship between HR and the accumulation of H_2_O_2_. Bolwell et al. (2002) indicated that H_2_O_2_ accumulation could be considered as an early signal molecule in the interaction between plant and pathogen and has direct antimicrobial effect by inducing gene expression and hypersensitive cell death (HCD). Plant defense responses mediated by signal transduction pathways (such as reactive oxygen species, SA, JA, and ET) lead to the reinforcement of cell walls, the production of PR proteins and antimicrobial metabolites, and even the HR that limits the development of the pathogen (Dangl and Jones, 2001; Hwang and Hwang, 2011). These findings were also similar to those observed in this study whereby the increased ion conductivity (**Figure 6A**) and H_2_O_2_ accumulation (**Figure 6B**), the up-regulation of the HR marker genes *NtHSR201* and *NtHSR203*, the JA associated genes *NtPR-1a/c* and *NtPR2*, and the ET synthesis-dependent genes *NtEFE26* and *NtAccdeaminase* (**Figure 6D**), as well as the antimicrobial action on tobacco pathogens *P. solanacearum* and *B. cinerea* (**Figure 7**) in *ScGluD*2-expressing leaves. Similarly, Mercado et al. (2015) demonstrated that transgenic strawberry plants expressing the beta-1,3-glucanase gene *bgn13.1* from *Trichoderma harzianum* in increased tolerance to crown rot disease. Liu et al. (2013) reported that the crude protein extract of transgenic tobacco lines that carrying the *PpGlu* gene from the fruit of *Pyrus pyrifolia* Nakai cv. Huobali inhibited the hyphal growth of the *Phomopsis* sp., *Alternaria* sp., and *Fusarium* sp.

In addition to the pathogen, various abiotic stresses can induce different expression patterns of plant beta-1,3-glucanases at the transcription or protein level (Akiyama and Pillai, 2001; Wu and Bradford, 2003). Choudhury et al. (2010) found that the transcript of β*-1,3-gluc* during ripening in banana fruit was strongly increased under ET treatment and partially induced by ABA stress. On the other hand, β*-1,3-gluc* was not induced under wound treatment, and was even negatively regulated under auxin, cold and white light treatments. The expression of *OsGLN1* from rice was up-regulated under ABA and drought stresses (Akiyama and Pillai, 2001). The levels of *GluB* transcripts were increased by MeJA and wounding in tomato seeds (Wu and Bradford, 2003). These findings suggest different functional properties among the members of beta-1,3-glucanases gene family in plants. Our group has also previously reported that the transcripts of *ScGluA1* were up-regulated under SA, MeJA, ABA, NaCl, CdCl_2_, and drought stresses, whereas *ScGluD1* was nearly down-regulated (Su et al., 2013). In this study, the expression level of *ScGluD*2 was partially increased under NaCl treatment, and steadily up-regulated upon ABA, H_2_O_2_, and CdCl_2_ stimuli (**Figure 4**). It is known that the broad-spectrum plant hormone ABA regulates plant development and physiology and responds to various environmental stresses such as salinity, drought, hypoxic and heavy metals contamination, and pathogen infection (Finkelstein et al., 2002; Pompeu et al., 2016). Scores of stress-responsive genes are up-regulated by ABA in osmotic imbalance (Ingram and Bartels, 1996). Oxidative stress is reported to be highly controlled by phytohormones (Pompeu et al., 2016). Cadmium (Cd) is one of the most toxic heavy metals which negatively affecting plant metabolism mainly by inducing oxidative stress (Cuypers et al., 2010; Pompeu et al., 2016). Han et al. (2012) indicated that ABA induced the tolerance of *T. aestivum* seedlings response to Cd stress. Our data suggests that ABA may be a signal molecule regulating oxidative stress and may play a role in the salt and heavy metal stress-induced stimulation of *ScGluD*2 transcripts. This is similar to the observations of Cheong et al. (2000) in which the expression of *SGN1* from soybean was strongly induced by a variety of defense-related signals including H_2_O_2_, wounding, the fungal elicitor from *Phytophthora parasitica* (Pmg), and the pathogen *P. syringae* pv. *glycinea* (Psg). However, the *SGN1* transcripts were barely induced upon SA, JA, and ET stresses (Cheong et al., 2000).

As is known, sugarcane is a highly polyploidy and aneuploidy crop (Scortecci et al., 2012). Selection of new resistant sugarcane clones with high yield and sucrose content needs a large segregation population of progeny and takes more than 10 years in conventional breeding (Dal-Bianco et al., 2012; Scortecci et al., 2012). Most modern sugarcane cultivars originate from crosses between a small number of original ancestor clones, resulting in a narrow genetic base (Jackson, 2005). These constraints create challenges for sugarcane genetic improvement (Lakshmanan et al., 2005; Scortecci et al., 2012). There are two major advantages for transgenic sugarcane, including the following: (i) sugarcane is an industrial crop and its sucrose production, which is chemically refined at 107°C, is the only consumer good that results in low-risk transgenic sugarcane; (ii) the short breeding process for its asexual reproduction to routinely multiply and maintain the genetically modified clones makes the transgenic technology using candidate genes an effective approach in transmitting commercially desirable traits to an elite variety (Falco et al., 2000; Henry and Kole, 2010; Dal-Bianco et al., 2012; Gómez-Merino et al., 2014). To date, a number of transgenic sugarcane transformed with genes expressing sugar improvement (Chong et al., 2007; Wu and Birch, 2007), herbicide resistance (Gallo-Meagher and Irvine, 1996; Manickavasagam et al., 2004), insect resistance to sugarcane stem borer (*Diatraea saccharalis* F.; Arencibia et al., 1997; Weng et al., 2006; Gao et al., 2016) and wooly aphid (*Ceratovacuna lanigera* Z.; Zhangsun et al., 2008), disease resistance to *Sugarcane mosaic virus* (SCMV; Gilbert et al., 2005) and *Xanthomonas albilineans* (Zhang et al., 1999), abiotic stress tolerance to drought (Zhang et al., 2006; Molinaria et al., 2007), and recombination protein of ER-targeted human cytokine protein GM-CSF (Wang et al., 2005) and aromatic hydroxybenzoic acid (pHBA; Petrasovits et al., 2007) have been successfully developed. Although traits of interest are being tested in various sugarcane cultivated countries, no commercial transgenic sugarcane line has been reported (Cheavegatti-Gianotto et al., 2011; Dal-Bianco et al., 2012; Ye et al., 2016), except for three drought-tolerant sugarcane transgenic events, NXI-1T, NXI-4T, and NXI-6T, which have been approved in Indonesia^8^ but without commercial cultivation.

The process of transformation has limitation because of its complex engineering system, involving the selection of receptor cells, exogenous gene, transformation methods, and screening system (Chen et al., 2011). As reported, there are several problems in the field of transgenic sugarcane research such as low transformation efficiency and transgene expression, single target gene and promoter, and the rarely investigated biosafety issue (Chen et al., 2011; Dal-Bianco et al., 2012; Scortecci et al., 2012). Therefore, the focus of future sugarcane transformation research should take into account the following aspects: (i) screening suitable recipient materials and optimizing the transformation efficiency, (ii) isolating new promoter regions (both constitutive and inducible) and vectors to improve the expression efficiency of the target gene, (iii) co-transforming multi-gene to achieve an elite transgenic sugarcane cultivar with a variety of traits at the same time, (iv) employing the security screening or no screening marking transformation method to make the transgenic sugarcane be more secure (Chen et al., 2011; Dal-Bianco et al., 2012; Scortecci et al., 2012; Gómez-Merino et al., 2014). Future developments would be expected to widen the application prospect of transgenic sugarcane. At present, although low transformation efficiency remains one of the major limiting factors in sugarcane production (Dal-Bianco et al., 2012; Scortecci et al., 2012; Gómez-Merino et al., 2014), the process of commercialization for transgenic sugarcane largely depends on mining for functional genes, particularly key functional genes that can significantly improve important agronomic or industrial traits. The findings of the present study suggest that elevated levels of *ScGluD*2 transcripts reflect a stress response. Whether this gene plays a direct causal role in defense against smut pathogen and/or salt and heavy metal stimuli needs further investigation. Besides, using model plant species systems that possess shorter life cycles and simpler genomes such as *Brachypodium distachyon* and *Setaria italica*, which belonging to *Gramineae* together with sugarcane, may be utilized as an alternative in future transgenic sugarcane experiments for the functional identification of new genes (Dal-Bianco et al., 2012).

## Conclusion

The present study showed that a *ScGluD*2 cDNA encoded a novel sugarcane acidic subfamily D beta-1,3-glucanase. It contained a X8 domain at the C terminus and was localized to the plasma membrane, indicating beta-1,3-glucan-binding function. The *ScGluD*2 expression was rapidly induced by smut pathogen infection at the early stage in sugarcane smut-resistant cultivar-*S. scitamineum* interaction, suggesting its involvement in the defense against the invasion of *S. scitamineum*. The transient overexpression and the antimicrobial action test in *N. benthamiana* aim to further but partly determine the function of ScGluD2 protein, which indicated that it is related to plant resistance to pathogen inoculations. In addition, we speculated its potential role in protecting sugarcane from salt and heavy metal stresses based on the observation that *ScGluD*2 was up-regulated by ABA, H_2_O_2_, NaCl, and CdCl_2_. Notably, *ScGluD*2 is differently regulated in comparison with sugarcane beta-1,3-glucanase genes *ScGluA1* and *ScGluD1* due to their different responses to adverse stimuli. These results revealed that *ScGluD*2 is a novel sugarcane stress-related gene involved in the defense response against smut pathogen infection and salt and heavy metal stresses.

## Author Contributions

YS, LX, and YQ conceived and designed the experiments. YS, ZW, FL, ZL, QP, and JG performed the experiments. YS analyzed the data and wrote the paper. LX and YQ revised the paper. All authors read and approved the final version of the paper.

## Conflict of Interest Statement

The authors declare that the research was conducted in the absence of any commercial or financial relationships that could be construed as a potential conflict of interest.

## Footnotes

1 http://www.ncbi.nlm.nih.gov/gorf/gorf.html

2 http://web.expasy.org/protparam/

3 http://www.cbs.dtu.dk/services/SignalP/

4 http://www.cbs.dtu.dk/services/TMHMM-2.0/

5 http://www.cbs.dtu.dk/services/NetNGlyc/

6 http://smart.embl-heidelberg.de/

7 http://psort.hgc.jp/form.html

8 http://www.isaaa.org/gmapprovaldatabase/advsearch/default.asp?CropID=27&TraitTypeID=Any&DeveloperID=Any&CountryID=Any&ApprovalTypeID=Any
