# Supplementary material for: Isolation and Characterization of ScGluD2, a New Sugarcane beta-1,3-Glucanase D Family Gene Induced by Sporisorium scitamineum, ABA, H2O2, NaCl, and CdCl2 Stresses
Source: Front Plant Sci. 2016 Sep 2;7:1348. doi: 10.3389/fpls.2016.01348 (PMC5009122; doi:10.3389/fpls.2016.01348)
Supplement: Supplementary file 1 [file Table_1.DOCX]

**Isolation and characterization of *ScGluD*2, a new sugarcane beta-1,3-glucanase D family gene induced by *Sporisorium scitamineum*, ABA, H_2_O_2_, NaCl, and CdCl_2_ stresses**

**Yachun Su, Zhuqing Wang, Feng Liu, Zhu Li, Qiong Peng, Jinlong Guo, Liping Xu*, Youxiong Que***

Key Laboratory of Sugarcane Biology and Genetic Breeding, Ministry of Agriculture, Fujian Agriculture and Forestry University, Fuzhou 350002, China

***Correspondences:** Liping Xu and Youxiong Que, Key Laboratory of Sugarcane Biology and Genetic Breeding, Fujian Agriculture and Forestry University, Ministry of Agriculture, No. 15 Shangxia Dian Road, Cangshan District, Fuzhou city, Fujian Province, 350000, P.R. China. E-mails: xlpmail@126.com, [queyouxiong@126.com](mailto:queyouxiong@126.com).

**Supplementary Table S1** **Nine sugarcane cultivars with different resistance to smut after identification in the field**

| **NO.** | **Variety** | | **Sugarcane smut incidence (%)** | **Resistance classification** | | **Resistance rating** |
| --- | --- | --- | --- | --- | --- | --- |
| 1 | YZ03-258 | 0.0 | | 1 | HR | |
| 2 | YZ01-1413 | | 4.5 | 2 | R | |
| 3 | LC05-136 | | 4.5 | 2 | R | |
| 4 | YT96-86 | | 5.0 | 2 | R | |
| 5 | GT02-467 | | 14.1 | 5 | MS | |
| 6 | ROC22 | | 14.5 | 5 | MS | |
| 7 | FN39 | 17.0 | | 5 | MS | |
| 8 | YZ03-103 | | 30.6 | 6 | S | |
| 9 | FN40 | | 33.1 | 6 | S | |

The smut resistance ratings in sugarcane according to Chao et al. (1990) and Xu et al. (2001) are as follows: 0-3%, HR; 4-6%, R; 7-9%, R; 10-12%, MR; 13-25%, MS; 26-35%, S; 36-50%, S; 51-75%, HS; 76-100%, HS. HR, highly resistance; R, resistance; MR, moderate resistance; MS, moderate susceptibility; S, susceptibility; HS, highly susceptibility. The data of sugarcane smut incidence comes from personal communication with Yingkun Huang.

**REFERENCES**

Chao, C.P., Hoy, J.W., Saxton, A.M., and Martin, F.A. (1990). Heritability of resistance and repeatability of clone reactions to sugarcane smut in Louisiana. *Phytopathology* 80, 622-626. doi:10.1094/phyto-80-622

Xu L. P., and Chen, R.K. (2001). Analysis of sugarcane segregating population and construction of pools resistant or sensitive to smut. *J. Fujian Agr. U.* 30, 153-157. doi:10.3321/j.issn:1671-5470.2001.02.006
