# Supplementary material for: Isolation and Characterization of ScGluD2, a New Sugarcane beta-1,3-Glucanase D Family Gene Induced by Sporisorium scitamineum, ABA, H2O2, NaCl, and CdCl2 Stresses
Source: Front Plant Sci. 2016 Sep 2;7:1348. doi: 10.3389/fpls.2016.01348 (PMC5009122; doi:10.3389/fpls.2016.01348)
Supplement: Supplementary file 2 [file Table_2.DOCX]

**Isolation and characterization of *ScGluD*2, a new sugarcane beta-1,3-glucanase D family gene induced by *Sporisorium scitamineum*, ABA, H_2_O_2_, NaCl, and CdCl_2_ stresses**

**Yachun Su, Zhuqing Wang, Feng Liu, Zhu Li, Qiong Peng, Jinlong Guo, Liping Xu*, Youxiong Que***

Key Laboratory of Sugarcane Biology and Genetic Breeding, Ministry of Agriculture, Fujian Agriculture and Forestry University, Fuzhou 350002, China

***Correspondences:** Liping Xu and Youxiong Que, Key Laboratory of Sugarcane Biology and Genetic Breeding, Fujian Agriculture and Forestry University, Ministry of Agriculture, No. 15 Shangxia Dian Road, Cangshan District, Fuzhou city, Fujian Province, 350000, P.R. China. E-mails: xlpmail@126.com, [queyouxiong@126.com](mailto:queyouxiong@126.com).


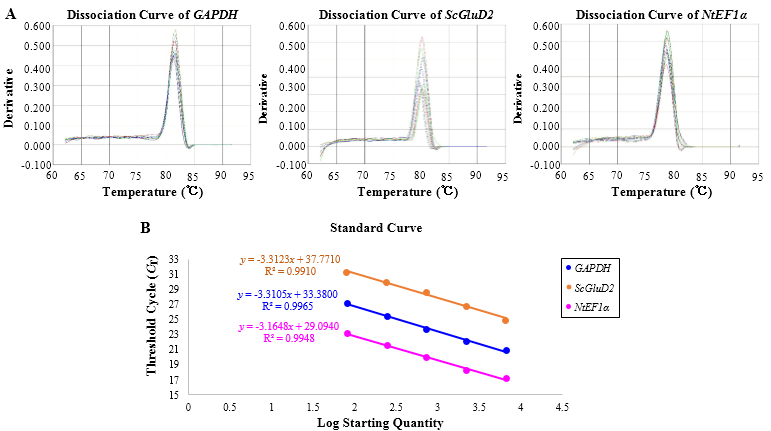


**Supplementary Figure S1 Fluorescence dissociation curves (A) and standard curves (B) of target gene *ScGluD*2 and internal control genes *GAPDH* in sugarcane, and *NtEF1α* in *Nicotiana benthamiana* by qRT-PCR analysis.** The untreated cDNA templates of Yacheng05-179 plantlet and *N. benthamiana* were used to determine the PCR efficiencies of *ScGluD*2/*GAPDH*, and *NtEF1α*, respectively. A series of 3-fold dilutions of cDNA (range, 3^4^ to 3^8^) was used as the template for qRT-PCR. The regression equations of *ScGluD*2, *GADPH*, and *NtEF1α*, which were calculated between the log cDNA dilution (*x*-axis) and threshold cycle (*C*_T_ value) (*y*-axis), were *y* = −3.3123*x* + 37.7710 (R^2^ = 0.9910, slope = −3.3123, E = 100.40%), *y* = −3.3105*x* + 33.3800 (R^2^ = 0.9965, slope = −3.3105, E = 100.48%), and *y* = −3.1648*x* + 29.0940 (R^2^ = 0.9948, slope = −3.1648, E = 107.00%), respectively.
